# Effect of Graded Substitution of Soybean Meal by *Hermetia illucens* Larvae Meal on Animal Performance, Apparent Ileal Digestibility, Gut Histology and Microbial Metabolites of Broilers

**DOI:** 10.3390/ani11061628

**Published:** 2021-05-31

**Authors:** Kristina Hartinger, Julia Greinix, Nathalie Thaler, Marco Antonio Ebbing, Nadia Yacoubi, Karl Schedle, Martin Gierus

**Affiliations:** 1 Department of Agrobiotechnology, Institute of Animal Nutrition, Livestock Products, and Nutrition Physiology (TTE), IFA-Tulln, University of Natural Resources and Life Sciences, 1190 Vienna, Austria; kristina.hartinger@boku.ac.at (K.H.); julia.greinix@students.boku.ac.at (J.G.); natalie.thaler@students.boku.ac.at (N.T.); marco.ebbing@boku.ac.at (M.A.E.); karl.schedle@boku.ac.at (K.S.); 2 Evonik Operations GmbH-Nutrition and Care, Rodenbacher Chaussee 4, 63457 Hanau-Wolfgang, Germany; nadia.yacoubi@evonik.com

**Keywords:** broiler chicken, *Hermetia illucens*, ileal digestibility, insects, performance, protein

## Abstract

**Simple Summary:**

Soybean meal (SBM) constitutes the major protein source in European poultry production, meaning a high dependency on imports and a reduced sustainability of produced meat. To cope with this challenge, alternative protein sources are needed, and insects are considered as a novel, alternative protein source in broiler nutrition. The objective of the present study was to evaluate the replacement of 15 or 30% of crude protein (CP) from SBM with *Hermetia illucens* (HI) defatted larvae meal CP regarding broiler performance, carcass traits, apparent ileal CP and amino acid (AA) digestibility, intestinal morphology, and microbial metabolites. The data showed impaired performance and lower ileal CP and AA digestibility with 30% substitution of CP from SBM with HI larvae meal CP. However, lower substitution, i.e., 15% substitution of SBM CP with HI larvae meal, for broiler feeds seems possible without impairment in animal performance and digestion variables and should be pursued in the future.

**Abstract:**

The usage of insects as an alternative protein source for broiler feeds may help to reduce the dependency on soybean meal (SBM) imports. Therefore, the present study aimed to evaluate the replacement of 15 (SL15) or 30% (SL30) of crude protein (CP) from SBM with *Hermetia illucens* (HI) defatted larvae meal regarding broiler performance, carcass traits, apparent ileal digestibility, intestinal morphology, and microbial metabolites. Concerning the performance, body weight was similar for the control (CON) and SL15, but lower for SL30 during all feeding phases. In addition, average daily feed intake was higher in SL15 and SL30 compared to CON in the starter phase, but this effect vanished during grower and finisher phase. The apparent ileal digestibility decreased for CP and some amino acids with increasing HI larvae meal in the diet. No or marginal alterations were observed for the intestinal morphometry as well as cecal microbial metabolites. In conclusion, partial replacement of 15% SBM CP with HI larvae meal in broiler diets without impairing animal performance or health seems possible. The growth suppression with 30% CP substitution may be caused by reduced apparent ileal digestibility but could not be clearly associated with adverse effects of hindgut fermentation or altered gut morphology.

## 1. Introduction

The production of broiler meat requires substantial amounts of grain feedstuffs [1] including immense quantities of soybean meal (SBM), the primarily used protein source in poultry production. One problem is that about 98% of crude protein (CP) originating from SBM and used for feed in the European Union (EU) in 2017/2018 was imported mainly from South America [2,3]. This illustrates the severe “protein gap” regarding production and demand of SBM and revealing the strong dependency on imports of protein-rich feedstuffs in the EU. Considerable effort has been made in finding alternative and more sustainable protein sources with low feed-food competition. In this respect, an interest in insects as food and feed has evolved [4,5]. Particular scientific research interest regarding suitability of insects in broiler feed has been aroused in the EU [6]. Currently only insect fat and live insects are permitted as feed for farmed animals, while non-hydrolyzed proteins are prohibited. Nevertheless, an exception was made in 2017, authorizing the use of seven insect species as feedstuff for aquaculture in the EU [7]. However, application of insects in pig and poultry feeds might soon be possible since a new risk profile from EFSA is expected in the near future.

Among about 2000 edible insect species worldwide, *Hermetia illucens* (HI), also known as black soldier fly, may possess a nutritional composition suitable for poultry nutrition as HI meal provides excellent apparent metabolizable energy corrected for nitrogen (AMEn) and digestible amino acids (AAs) to broilers [8,9,10,11]. However, the nutritive value of HI is highly dependent on the rearing substrate [12,13] as well as the developing state of larvae at harvest [14]. Nevertheless, HI larvae are characterized by a high CP concentration ranging from 37 to 63% in dry matter (DM), combined with a crude fat content varying between 7 and 39% in DM. As the high fat concentration may impair digestibility, processed insect products, such as fat-reduced (protein) meals, are of greater importance for poultry nutrition [10,15].

Suggestions about the optimal inclusion rate of HI larvae meal in broiler feeds are yet inconsistent with recommendations ranging from a maximum of 15 to 50% in the diet [6,16,17,18,19]. Higher dosages of HI seem to negatively affect the animal performance [17], which may be related to the high amount of N as non-amino acid N in larvae compared to plant protein sources [20]. Based on these foregoing observations, it seems that HI larvae meal in low rates can be included in poultry diets without hindrance. However, to the authors’ best knowledge, limited digestibility data of different larvae meals are available, and the results are rather inconsistent. In addition, the mechanisms involved in the digestion of HI larvae in diets of broilers is poorly documented. Regarding this, digestion and absorption of ingested feed, as the main functions of the intestine, have a direct or indirect impact on animal health [21], and for the effective nutrient digestion and absorption, the absorptive epithelium of the small intestine is of particular importance [22]. Its organization in villi-crypts units aims to optimize nutrient absorption by maximizing the absorptive area [23]. Therefore, we expected a correlation for performance and gut morphology of the birds when increasing HI larvae meal in the diets.

Next to morphological changes in the gut epithelium, microbial activity in the gut may show differences in the nutritive value of diets. As already known, the ceca are the main site of microbial fermentation in broilers, due to their high bacterial density being 100–1000-fold higher compared to ileal digesta [24]. Microbial metabolites are generated and arise from protein and carbohydrate fermentation. The generated microbial metabolites from protein fermentation comprise amines, indoles, phenols, cresols, and ammonia and altogether may have adverse effects on broiler growth and performance, when present in high concentrations [25]. Apart from microbial and endogenous protein, also resistant protein of dietary origin and firmly bound nitrogen, like the N present in chitin, flows into the ceca. Hence, the amount of ileal undigested protein entering the ceca is determined by the ileal digestibility of dietary protein. This means the higher the digestibility, the lower the amount of resistant dietary protein entering the ceca and, therefore, also the putrefactive bacterial fermentation [24]. It is therefore of particular interest to investigate the cecal fermentation processes and compare to apparent ileal digestibility (AID) data to gain knowledge of the nutritional-physiological background of larvae meal digestion.

Therefore, the present study aimed to investigate the suitability and optimal inclusion level of defatted HI larvae meal by substituting 15 or 30% of CP from SBM with HI defatted larvae meal, corresponding to 4–10% HI larvae meal inclusion in the diets. Additionally, it was an objective to generate AID data to enable better assessment of nutrient quality of HI larvae meal. We hypothesized that, based on equal ileal digestibility of HI larvae meal and SBM, not only substituting low, but also higher amounts of SBM CP will lead to similar broiler performance, without impairments on gut morphology or alteration in microbial hindgut fermentation.

## 2. Materials and Methods

The feeding trial was approved by the Federal Office for Food Safety (Austria) according to § 10 Abs 1 Futtermittelgesetz 1999, BGBl. I Nr. 139/1999 (FMG), with the reference number BAES-FMT-FV-2018-0001.

### 2.1. Birds, Housing, and Diets

In total, 216 chickens 1 day old (Ross 308) of both sexes with an initial body weight (BW) of 40.3 g (±0.41 g) were purchased from a commercial local hatchery. The trial was carried out at a poultry research station rented by the University of Natural Resources and Life Sciences, Vienna, Austria, and housing of animals on wood shavings as litter material was carried out under compliance with the 1st regulation of keeping of animals (BGBl. II Nr. 485/2004). The average ambient temperature at the beginning of the study was 29 °C and was gradually decreased to 20 °C until the end of the experiment. The lighting schedule was 18 h light, 6 h dark. In order to receive similar mean weights per pen, animals were weighed at day 1 and correspondingly assigned to treatments. Thereby, birds were allocated to 18 pens with 12 animals each, resulting in 6 replicates per treatment.

All diets were calculated to meet the Breeder’s nutritional specifications [26] within a three-phase feeding program: starter diet was fed from day 1 to day 14, grower diet was fed from day 15 to day 28, and finisher diet was fed from day 29 to day 36. Due to scarce and inconsistent data concerning larvae meal digestibility, the present diets were calculated on the basis of Ross 308 Broiler Nutrition Specifications (2019) [26] of total AA. The control diet (CON) was based on corn and SBM. For calculation of the two experimental diets, the amount of CP supplied by SBM in the respective control diet of each phase was replaced in graded levels (15, 30%) by CP of HI larvae meal. *Hermetia illucens* larvae were reared on wheat bran, cracked rye, water, and fat-protein stillage. Following drying at 80 °C, the larvae were partly defatted with a screw press and afterwards ground into a meal. The composition of HI larvae meal is shown in Table 1. Thus, three different treatments were finally prepared, which are referred to as CON, substitution level (SL) 15 (SL15), and SL30. During all phases, diets were calculated to be both iso-energetic and iso-nitrogenous (Table 2), and diets were provided for ad libitum consumption. Moreover, diets were balanced for AA according to the Breeder’s nutritional specifications. All diets were expanded before pelleting. The starter diet was fed in crumbled form (granulation gap 1.7 mm), whereas grower (2.3 mm) and finisher (2.8 mm) diets were offered as pellets. Titanium dioxide was administered (3 g/kg fresh matter) to finisher feeds (29–35 d) prior to pelleting as external marker to determine AID. Animals had free access to water during the whole experiment.

### 2.2. Performance Parameters

Animal BW was determined pen wise on days 1, 14, and 28 and individually on day 35 for calculation of the performance parameters average daily gain (ADG), average daily feed intake (ADFI), and feed conversion ratio (FCR). Parameters were determined for each feeding phase as well as the overall experiment. Mortality was recorded as it occurred, and dead birds were weighed to determine the losses. Feed conversion ratio was calculated from ADFI and ADG.

### 2.3. Carcass Traits

At the end of the experiment (day 36), all broilers were weighed individually, stunned, and killed by bleeding. Subsequently, the following traits were collected, and weights recorded: dressing, eviscerated carcass (as the weight of the slaughtered broiler without blood), feather, giblets and intestinal tract, chilled carcass (as the weight of the carcass after 16 h of storage at 3 °C in a cooling chamber), carcass for grilling (as the carcass chilled without head, neck and legs at the hock joints), giblets (heart, liver, stomach), and abdominal fat.

### 2.4. Sample Collection

Directly after slaughtering, the intestinal tract was removed and opened from four representative broilers per pen (*n* = 72), i.e., two males and two females closest to the median of BW. Digesta from two gut sections (ileum (Meckel’s diverticulum until colon) and ceca (whole contents of both ceca)) were collected. To obtain enough digesta, homogenously mixed samples of four animals were pooled per pen, put into narrow mouth bottles, and immediately frozen at −20 °C until further analysis.

Samples for histological analysis were taken from two representative broilers (one male and one female) per pen (=36 animals), which were already taken for digesta sampling. Tissue samples were taken from the jejunum, halfway between the duodenum and the Meckel’s diverticulum, and the ileum 3–6 cm proximal to the ileocecal junction. Samples were washed thoroughly with ice-cold phosphate-buffered saline to remove the entire digesta content, embedded in slotted cassettes, and immersed in 4% paraformaldehyde for 48 h.

### 2.5. Chemical Analyses of Feed and Digesta Samples

Ileal digesta samples were thawed at 4 °C and freeze-dried. All samples were ground through a 1 mm sieve and homogenized. Caeca digesta samples were analyzed in fresh matter.

The proximate composition of all diets and HI larvae meal was analyzed in duplicate according to the standard procedures [27]: dry matter (DM; method no. 3.1.4), ash (CA; method no. 8.1.1), ether extract (EE; method no. 5.1.1), ether extract after acid hydrolysis (EEh; method no. 5.1.2), and crude fiber (CF; method no. 6.1.2). Nitrogen content of the diets was analyzed using Dumas combustion method (DuMaster 480, Büchi AG, Flawil, Switzerland) (method no. 4.1.2) [28] and multiplied by 6.25 to calculate CP concentration. Acid-detergent insoluble nitrogen (ADIN) was measured according to Licitra et al. [29]. Additionally, feed samples were wet-ashed in a microwave oven (CEM Mars 6, CEM Corp., Matthews, NC, USA) to analyze Ca, Na, and K by flame atomic absorption spectrophotometry (AAnalyst200, Perkin Elmer Inc., Massachusetts, USA), and P photometrically (Tecan Group Ltd., Männedorf, Switzerland) using the vanado-molybdate method at 436 nm [27] (method no. 10.6.1).

Feed and digesta samples were also analyzed for titanium dioxide (TiO_2_) concentration as described by Leone et al. [30]. Briefly, 0.5 g of sample was weighed and, after addition of a catalyst tablet, digested in 25 mL concentrated sulphuric acid at 400 °C for 115 min on a block digesta. After removing and cooling the tubes, digestion was decanted to a volumetric flask, and the volume was made up to 100 mL with distilled water. Following filtration, 5 mL of each sample was mixed with 1 mL 1M sulphuric acid and 1 mL hydrogen peroxide (300 mL/L). Subsequently, mixtures were measured at 405 nm using a UV–vis spectrophotometer (UV-1800, Shimadzu Corp., Kyoto, Japan) and compared to a titanium sulphate standard. The gross energy (GE) content in feed was determined by bomb calorimetry (IKA C 200, IKA Werke GmbH & Co. KG, Staufen, Germany). Total AA analyses of finisher diets and ileal digesta samples were determined by ion-exchange chromatography with post-column derivatization with ninhydrin, as described in detail by Figueiredo-Silva et al. [31]. The AA composition in insect meals was provided by the manufacturer, and the total content was used for diet formulation.

### 2.6. Microbial Metabolites

The concentrations of biogenic amines in cecal digesta were analyzed according to Saarinen [32] using reverse-phase HPLC (Waters 2695e Separations Module, Waters, MA, USA). A RP-18 column (InertClone™ 5 μm ODS (2) 150 Å, 250 × 4.6 mm, Phenomenex, Torrance, CA, USA) was used, and the detection was performed by a UV detector (Waters 2489 UV-visible detector, Waters, Milford, MA, USA). Data calculation was done by the software Empower 3 (Waters, Milford, MA, USA). For eluents 1 and 2, 0.1 M ammonia-acetate buffer (pH 5) (Carl Roth GmbH + Co. KG, Karlsruhe, Germany) and acetonitrile (HPLC grade; Carl Roth GmbH + Co. KG, Karlsruhe, Germany) were used, respectively. For determination of ammonia and total lactic acid content, approximately 1.0 g of digesta sample was vortexed with 1.0 mL perchloric acid (1 M), afterwards it was allowed to settle for 10 min, and then 8 mL of double-distilled water was added and vortexed again. Afterwards, samples were placed on a shaker (POLYMAX 1040, Heidolph Instruments, Schwabach, Germany) for 1 h and subsequently centrifuged for 10 min at 3215× *g* (Centrifuge 5810R, Eppendorf, Wesseling-Berzdorf, Germany). The supernatant was immediately stored in 2 mL tubes at −20 °C. Thawed samples were centrifuged at 12,066× *g* for 5 min (Minispin, Eppendorf, Wesseling-Berzdorf, Germany), and a supernatant fluid was used for the further analysis of lactic acid and ammonia.

To analyze the ammonia concentration, the samples were diluted, and a mixture of 0.5 mL of salicylate-nitroprusside color reagent (blend of equal parts of sodium hydroxide 0.3 M, ddest. H_2_O and salicylate-nitroprusside solution) and 0.25 mL of dichloroisocyanurate solution (0.050 g dichloroisocyanurate dissolved in 50 mL ddest. H_2_O) was prepared; 1.0 mL of sample extract or standard solution (Ammonia standard solution ROTI^®^Star, Karlsruhe, Germany) was added immediately for a proper coloring reaction. Afterwards, samples were incubated for 1.5 h in the dark at room temperature, and subsequently the concentration of ammonia was analyzed spectro-photometrically (Tecan Austria GmbH, Grödig, Austria) at 660 nm.

Total lactic acid content in cecal digesta was analyzed according to the procedure of Pryce et al. [33] with slight modifications regarding sample preparation and amount of reagent. Briefly, 100 μL sample or standard solution (Lithium L-lactate, Sigma-Aldrich, Steinheim, Germany) and 3.9 mL of precipitating reagent were mixed and centrifuged for 5 min at 3215× *g*. The following procedure was carried out with 0.5 mL of generated supernatant liquid, 3.0 mL sulphuric acid, and 50 µL p-hydroxybiphenyl. Absorbance at 565 nm was read using a spectrophotometer (Tecan Austria GmbH, Grödig, Austria).

### 2.7. Histomorphology

Gut tissue samples were dehydrated and embedded in paraffin wax blocks, sectioned at 5 μm thickness using a microtome (Leica RM2255, Leica Biosystems GmbH, Wetzlar, Germany), and mounted onto glass slides (Menzel-Gläser Superfrost-Plus, Thermo Scientific, Braunschweig, Germany). Afterwards, sections were stained (Leica Auto-Stainer XL ST5010, Leica Biosystems GmbH, Nussloch, Germany) following the standard protocol for Alcian blue-periodic acid–Schiff (AB-PAS). All morphometric indices examined were made on six well-orientated villi and crypts, respectively. Villus height was measured from the tip of the villus to the villus-crypt axis, villus width (Vw) (at the villus-crypt axis), villus area as cross-sectional area of a villus measured above the villus-crypt axis, and crypt depth from the base of the villus to the submucosa. Furthermore, the villus height-to-crypt depth ratio was calculated. Goblet cells were counted in six villi and are expressed as number of cells per 200 µm of villus epithelium. Furthermore, the thickness of the submucosa and muscularis circularis was determined in six randomly selected points. For visualization, a light microscope (Leica DM 6000 B, Leica) and the software Leica Application Suite (Leica, Version 4.13) were used.

### 2.8. Calculations

Feed conversion ratio was calculated according to the following equation:

$$FCR=\frac{Average\;daily\;feed\;intake}{Average\;daily\;gain}$$

The following equation was used to calculate the AID:

$$AID\;\%=\{1-[\left(\frac{TiO_{2}\;\%\;diet}{TiO_{2}\;\%\;digesta}\right)\times \left(\frac{CP\;or\;Amino\;acid\;\%\;digesta}{CP\;or\;Amino\;acid\;\%\;diet}\right)\}\times 100$$

### 2.9. Statistical Analyses

The general model was:

Y_ij_ = μ + Τ_i_ + e_ij_

where Y_ij_ is the dependent variable, μ is the overall mean, T_i_ is the treatment effect, and e_ij_ is the residual error, calculated using the Mixed procedure in SAS (Version 9.4; SAS Inst. Inc., Cary, NC, USA).

Pens are considered as the experimental unit for performance, AID and microbial metabolites, and animal for carcass yield and histological parameters. Significance was defined at *p* ≤ 0.05 and a statistical trend at *p* ≤ 0.10. Results are expressed as least-squares means, and differences between the least-squares means were tested post hoc using Tukey’s test.

## 3. Results

### 3.1. Animal Performance

Results of performance for starter, grower, finisher, as well as the entire experiment are shown in Table 3. Mortality during the entire experimental phase was 6.5%, accounting for one dead bird in CON and SL15, respectively, and 12 dead birds in SL30. The mortality in each feeding phase is given in Table 3. In the starter phase, HI larvae meal inclusion had an effect (*p* < 0.001) on BW, ADFI, ADG, and FCR. Considering BW and ADG, CON and SL15 were not different, but superior compared to SL30. In contrast, regarding ADFI, SL15 and SL30 were on the same level, but higher than CON. Feed conversion rate was best for CON, but it did not differ in SL15 and SL30.

In the grower phase, BW (*p* < 0.001), ADFI (*p* < 0.029), and ADG (*p* < 0.001) were higher in CON and SL15 compared to SL30. No differences caused by HI larvae meal were detected regarding FCR in the grower phase.

In the finisher phase, BW was higher (*p* < 0.001) in CON and SL15, compared to SL30. Additionally, CON showed (*p* = 0.025) higher ADFI compared to SL30, with SL15 being intermediate. FCR was improved in SL30 compared to CON and SL15. The FCR tended to be better (*p* = 0.079) for SL30 compared to CON.

Concerning the entire duration of the experiment, ADG was (*p* = 0.001) decreased in SL30 compared to CON and SL15. No statistically significant alterations were observed for ADFI and FCR for the overall experiment.

### 3.2. Carcass Traits and Weight of Giblets

The following carcass traits were affected by HI larvae meal inclusion: eviscerated carcass, carcass chilled, carcass for grilling, and abdominal fat (*p* < 0.001) (Table 4). All the latter were highest for CON and SL15 but decreased in SL30. Moreover, there was a trend (*p* = 0.075) for higher dressing, with SL15 being tendentially higher than SL30, and CON as intermediate. Birds fed SL15 had a higher liver weight (*p* = 0.03) compared to CON and SL30. In contrast, gizzard weights decreased from CON to SL30 (*p* = 0.001). Increasing HI larvae meal inclusion tended to affect breast weight (*p* = 0.057) and was highest for SL15 and SL30 compared to CON.

### 3.3. Apparent Ileal Digestibility

Apparent ileal digestibility values of CP and AA are shown in Table 5. The AID of CP was affected (*p* = 0.014) by HI larvae meal with CON having a 5% higher digestibility compared to SL30. However, SL15 was intermediate, not differing from either CON or SL30. As far as AAs are concerned, the AID of leucine and cysteine was affected by treatment (*p* = 0.016 and *p* = 0.007, respectively) with SL15 and SL30 being lower than CON. The HI larvae meal inclusion also affected histidine (*p* = 0.031), asparagine (*p* = 0.044), glycine (*p* = 0.045), and serine (*p* = 0.041), where AID of SL30 was lower compared to CON, with SL15 as intermediate. Apparent ileal digestibility of isoleucine was decreased (*p* = 0.008) in SL30 compared to CON and SL15.

### 3.4. Intestinal Morphometric Indices

Results of the morphological measurements are presented in Table 6. In the jejunum, a trend (*p* < 0.1) was observed for Vw. In the ileum, no differences caused by HI larvae meal were detected regarding the morphometric indices under investigation.

### 3.5. Microbial Metabolites

In response to an increase in SBM CP substitution by HI larvae meal from 0 to 30%, the cecal concentrations of measured biogenic amines were not affected (Table 7). However, ammonia concentration was affected (*p* = 0.019) by HI larvae meal inclusion and was higher for CON and SL15 compared to SL30. No statistically significant differences were observed for the concentration of lactic acid.

## 4. Discussion

### 4.1. Animal Performance

The present experiment tested the suitability of low amounts of HI larvae meal as replacement for SBM CP in broiler feeding. The trial revealed impaired animal performance in terms of ADG for the complete experimental phase when 30% CP of SBM was replaced by HI larvae meal, which, depending on the feeding phase, corresponded to 4–10% HI larvae meal inclusion. Apart from that, also mortality was higher for SL30 compared to CON and SL15, i.e., 12 birds in SL30 and 1 in CON and SL15, respectively. One potential reason for the impaired animal performance in SL30 might be an immature gastrointestinal tract of broilers, i.e., in the starter phase, since no reductions in animal performance were observed when substituting up to 100% of SBM by HI larvae meal in laying hens [34]. Therefore, suitability of HI larvae meal feeding may depend on broiler age and may improve in the finisher phase, i.e., when the gastrointestinal tract is matured. In addition, differences in protein requirements may also play a role in the variation observed between broiler and laying hens.

The present observations on animal performance are in line with Dabbou et al. [17], who fed increasing levels of HI larvae meal and monitored improved performance parameters with a maximum at 10% HI larvae meal inclusion, but decreased animal performance when HI larvae meal accounted for 15% in the diet. Likewise, the present findings also match with a recent meta-analysis on the effects of insects as feed ingredients in poultry feed, which found decreased ADG if processed or unprocessed insects accounted for more than 10% of the diet [6]. However, other experiments reported 50% or even more SBM CP replacement by HI larvae meal (accounting for up to 25% HI larvae meal inclusion in the diet) with similar or better animal performance between the control and larvae-fed groups, although this was only true when AA requirements were met or even exceeded by addition of synthetic AA [19,35]. Additionally, BW of CON in the present study was superior compared to the official AVIAGEN Ross308 performance objectives as well as to the SBM-fed control groups in other studies [17,19], elevating the benchmark for comparing the experimental groups in our study. However, it is noteworthy that SL15 was able to keep up with that high benchmark in several performance parameters, thus confirming our hypothesis and showing the general feasibility of including HI larvae meal in lower concentrations, i.e., 15% of CP.

An interesting observation was a higher ADFI for SL15 and SL30, which was only observed in the starter phase. Similar results for the starter phase were also observed during feed choice trials by Cullere et al. [16] and Dabbou et al. [17]. Moreover, higher ADFI might have been from the birds attempting to compensate for lower nutrient and energy yield from the diet, although this was not reflected in BW, which was lower in SL30 during the starter phase. The still lower BW may also be the reason why the contrary ADFI was observed in grower and finisher phases. Gillette et al. [36], for example, found that illness or other unpleasant post-ingestive effects will be associated with visual characteristics. Birds learn avoidance towards those diets, and such effects might hold true for SL30. Provided that intolerances due to an immature gastrointestinal tract at the beginning of the experiment subsequently affected broiler ADFI and eventually also BW, it may be interesting to investigate higher HI inclusion rates solely in the finisher phase in future studies. Another possible explanation for differing ADFI might be the ability of chickens to select between diets differing in protein quality [37]. Since chickens had no choice in the present experiment, and gross energy (in starter and finisher phase), ileal CP, and AA digestibility decreased from CON to SL30, they may have eventually consumed less feed in response. Concerning the FCR of SL30 birds in the finisher phase, it seems unexpected that these birds have a lower BW, but tended to have a better FCR than CON or SL15. However, this observed circumstance may have likely been caused by a retention of water in the body. The SL30 birds partly showed signs of ascites at slaughter (0%, 5.6%, and 8.3% for CON, SL15, and SL30, respectively), which then can lead to an apparently higher mean BW in SL30 that, however, is not an increase of body mass but retention of water.

### 4.2. Carcass Traits and Weight of Giblets

Apart from zootechnical performance, from an economic point of view, it is of utmost importance that alternative protein sources do not negatively affect carcass traits. The present experiment showed negative responses of eviscerated carcass, carcass chilled, and carcass for grilling to SL30, while SL15 was similar to CON. This contrasts with Pieterse et al. [38] and Altmann et al. [39], who found no differences when feeding up to 15% HI larvae meal on carcass characteristics and higher carcass weight, respectively. However, it has to be noted that present values for carcass chilled and dressing were higher for all treatment groups compared to the experiment of Pieterse et al. [38], slaughtering at the same age.

### 4.3. Apparent Ileal Digestibility of CP and AA

To determine the nutritional value and especially protein quality of feedstuffs for poultry, the ileal AA digestibility is a central measure. A decrease in AID of CP with increasing larvae meal inclusion was observed, which was also apparent for several AAs. This finding is in line with the observation of de Marco et al. [9], who found lower AID coefficients in full-fat HI meal compared to SBM and other protein sources. However, AID of most of the AAs were equal for CON and SL15 in the present experiment, which matches with Schiavone et al. [8], who concluded that AID coefficients of HI larvae meal are similar to that of SBM. It must be noted, however, that in the present experiment both the aminogram and the digestibility were determined for the diets and not only the HI larvae meal. Nevertheless, since HI larvae meal was the main varying factor, observed changes in digestibility may most likely be attributed to it.

It has often been speculated [8,9,17] that one reason for a decrease in AID in HI diets might be the structural components in the exoskeleton of arthropods, namely chitin. The natural polysaccharide chitin, naturally occurring as ordered crystalline microfibrils, fulfils many functions to guarantee stability of the insect [40]. Although Hossain and Blair [41] stated that birds would possess sufficient endogenous chitinase activity that enables the digestion of chitin, which is supported by recent mRNA expression-based findings on the specific level of the chitin-degrading enzyme acidic chitinase [42], HI-derived chitin may still have contributed to the impaired digestibility. Regarding this, the measurement of ADIN (Table 1), as nitrogen bound to ADF, could be used as indicator for high proportions of chitin in larvae meal [43]. With approximately 75 g/kg CP, the value obtained in the present study is double as observed in corn (30 g/kg CP) and more than three times higher as in soybean meal (20 g/kg CP), as observed by van Soest [44]. In addition, heat damage seems to be also identified by ADIN determination. In this case, next to the chitin content, heat damage by processing of larvae meal cannot be excluded.

Moreover, Janssen et al. [20] see an overestimation in the conventional nitrogen-to-protein conversion factor of 6.25, suggesting the use of a specific conversion factor of 4.76 for HI larvae. This factor is based on their finding that 13–26% of total nitrogen in insects constitute non-protein nitrogen (e.g., chitin, chitosan, nucleic acids, inorganic N), and 3.0–6.8% of total nitrogen is bound in chitin, therefore overestimating the true protein content of insect meals. This is supported by a study of Nery et al. [45], who calculated the CP digestibility using conventional and newly proposed nitrogen-to-protein conversion factors and proposed that it is more accurate to use a specific nitrogen-to-protein conversion factor for diets containing insect meals. Taking these considerations into account, bioavailable nitrogen is falsely overestimated, and the reduced AID of CP in the 30SL diets may therefore partly be ascribed to this overestimation. Consequently, instead of assessing the AID of CP, it seems more reasonable to determine the AID of AA to assess HI protein quality. Indeed, AID of several AAs were reduced in the SL30 treatment, which could explain the reduced performance characteristics of broilers in this feeding group. However, one cannot exclude the possibility that AID of AAs were distorted by the influence of endogenous losses, which are not considered when using the present method. Changes in endogenous AA losses could have been caused, for example, by the significant differences in ADFI or differences in CP content of the diets [46].

Generally, it is urgently mandatory to generate more digestibility data for HI larvae meal since existing data in part differ considerably, as becomes obvious when comparing the present results with those of Schiavone et al. [8] and de Marco et al. [9]. Future research should focus on investigating ileal digestibility of AA and CP in HI larvae meal since this is a prerequisite for diet formulations and, so far, is missing knowledge. Therefore, it is necessary to explicitly characterize the used HI larvae in terms of larval instar, rearing substrate and processing since these aspects substantially affect larval nutrient composition and digestibility.

### 4.4. Histological Parameters

Regarding the effective nutrient digestion and absorption, the absorptive epithelium of the small intestine is of particular importance [22]. The experiment has shown that HI larvae meal inclusion only marginally affected the intestinal morphology. Birds of SL15 tended to have a greater Vw than CON, with SL30 as intermediate. The lack of alterations in gut integrity was rather unexpected since considerable differences between treatment groups were shown at the zootechnical performance level. Additionally, the absence of histological alterations in the gut tissues contradicts the decreased ileal CP digestibility as intestinal nutrient absorption rate is one factor inducing changes in villus morphology [47]. Similarly, Chu et al. [48] and Biasato et al. [49] did not find changes in gut morphology in laying hens fed up to 9% HI full-fat meal and free range chickens fed 7.5% full-fat *Tenebrio molitor* meal in their diets, respectively. Our findings contrast with the observations of other authors, who determined higher crypt depth and decreased villus height/crypt depth ratio, when replacing SBM up to 100% by HI larvae meal [17,19,50]. Based on our findings, and despite reduced animal performance, replacement of CP from SBM by HI larvae meal CP did not impair gut integrity when fed in low amounts up to 30% SBM CP substitution.

### 4.5. Microbial Metabolites in the Caecum

Regarding biogenic amines as products of AA decarboxylation, no alterations in their digesta concentrations could be observed between the treatment groups. Considering the lower AID of several AA for SL30, compared to CON and SL15, thus likely increasing the amount of undigested protein flowing to the hindgut, more microbial activity in the hindgut was expected. A similar unexpected observation was made by Qaisrani et al. [51], who fed different levels of low digestible rapeseed meal at expense of SBM. The authors were expecting higher biogenic amines in the rapeseed meal fed group, but Qaisrani et al. [51] observed higher cecal concentrations of biogenic amines in the SBM group. Moreover, the decreasing ammonia content in digesta determined for the HI larvae inclusion levels in our study coincides with Bryan et al. [52] and Qaisrani et al. [51]. These authors also observed higher ammonia levels in digesta with high digestible protein compared to low digestible protein, supporting our observations.

Several factors could have contributed to these observations in our study. First, a higher amount of undigested but potentially fermentable carbohydrates such as fiber may have flowed to the hindgut and be preferentially used by the microbiota as energy source, instead of protein. Such an effect, meaning a shift from protein fermentation to carbohydrate fermentation, was shown by Pieper et al. [53] in pigs, showing a reduced formation of biogenic amines and ammonia with increasing fermentable carbohydrates in the diet. This would explain the numerically lower biogenic amine formation in SL30 (crude fiber = 36.2 g/kg DM) compared to CON (crude fiber = 32.5 g/kg DM). However, lactic acid concentration, as one parameter for carbohydrate fermentation, was only numerically increased in high HI larvae meal groups. Secondly, the higher concentration of ADIN in HI larvae meal (Table 1) may help to explain these results. Since ADIN is not only regarded as indigestible for the host but also for microorganisms [44], HI larvae meal protein might not only have escaped host digestion in the small intestine, but also the microbial fermentation in the caecum. Thereby, the lower cecal ammonia concentrations observed in SL15 and SL30 substantiate this assumption. Consequently, our findings suggest that several factors have led to the microbial metabolite concentrations observed in the hindgut of broilers, which, however, could not be univocally designated by the measured parameters. Therefore, determination of cecal SCFA concentrations and cecal microbiome analysis in future experiments may help to monitor potential changes in hindgut fermentation patterns, e.g., shifts from protein to carbohydrate fermentation.

## 5. Conclusions

The present study provides detailed information on the effects of substituting different levels of CP from SBM with HI larvae meal in broiler feeding. The observed reductions in animal performance with increasing HI larvae meal inclusion may be ascribed to a misjudgment of both CP and several AA availabilities, as reflected by their lower AID. In conclusion, it seems likely that the lower ileal CP and AA digestibility resulted in the impaired performance with 30% substitution of CP from SBM with HI larvae meal CP. However, the impaired performance could not be conclusively associated to adverse effects of hindgut fermentation or altered gut morphology. The observed results of feeding HI larvae meal as an alternative protein source in broiler diets reflect the trend of previous studies, which showed that SBM can yet not be fully replaced by HI larvae meal without impairing animal performance. However, in smaller quantities, i.e., by replacing 15% of CP, HI larvae meal did not reduce performance characteristics or other variables investigated and may therefore be suitable as an option to reduce SBM need as protein source in broiler diets.

## Figures and Tables

**Table 1:** Crude nutrients of the defatted larvae meal (g/kg DM).

Item	HI Larvae Meal
Dry matter (g/kg as fed basis)	957
Crude protein	637
Acid-detergent insoluble nitrogen (g/kg CP)	74.7
Ash	119
Crude fat	62.7
Crude fiber	87.3
Phosphorus	12.2
Calcium	23.4
Sodium	1.2
Potassium	17.0

**Table 2:** Ingredient (g/kg fresh matter) and analyzed nutrient composition of experimental diets.

Items	Starter Phase (1–14 d)			Grower Phase (15–28 d)			Finisher Phase (29–35 d)		
	CON	SL15	SL30	CON	SL15	SL30	CON	SL15	SL30
Corn	508	526	544	553	562	572	565	583	600
Soybean meal (48% CP)	408	338	268	353	297	241	330	275	219
HI larvae meal	0.0	50.0	100	0.0	43.9	86.8	0.0	40.5	80.9
Soybean oil	27.0	21.4	15.8	37.3	33.6	29.9	52.8	47.7	42.4
Dicalcium phosphate	14.3	12.4	10.5	13.0	10.4	7.8	10.0	7.2	5.7
Grass meal	10.0	16.2	22.0	11.1	17.1	22.7	10.2	12.0	14.3
Feed limestone	10.6	11.8	13.0	10.0	12.1	14.5	10.2	11.0	12.1
Mineral and vitamin premix ^1^	10.0	10.0	10.0	10.0	10.0	10.0	10.0	10.0	10.0
Salt	4.0	4.1	4.3	4.0	4.0	4.0	4.0	4.0	4.0
L-methionine (99%)	3.0	3.0	3.0	2.2	2.2	2.2	2.0	2.1	2.2
BIOLYS ^2^	3.0	4.3	5.7	3.5	4.3	5.1	1.5	2.5	3.6
L-threonine	1.0	1.2	1.4	1.0	1.2	1.4	0.7	0.8	0.9
L-valine	0.0	0.0	0.1	0.8	0.8	0.8	0.0	0.0	0.0
Arginine	0.0	0.6	1.2	0.0	0.4	0.8	0.0	0.7	1.4
Choline chloride (60%)	0.5	0.4	0.4	0.5	0.4	0.4	0.5	0.4	0.4
Coban 200 ^3^	0.5	0.5	0.5	0.5	0.5	0.5	0.0	0.0	0.0
Optiphos ^4^	0.1	0.1	0.1	0.1	0.1	0.1	0.1	0.1	0.1
Titanium dioxide	0.0	0.0	0.0	0.0	0.0	0.0	3.0	3.0	3.0
Analyzed nutrient composition (g/kg DM)									
Dry matter (g/kg as fed basis)	893	901	902	897	902	905	898	900	906
Ash	76.5	78.1	77.4	67.8	71.0	73.8	71.3	69.3	68.8
Crude protein	271	272	269	246	245	249	225	238	240
Ether extract ^5^	65.0	67.7	61.7	83.2	79.2	75.5	99.3	97.3	91.6
Crude fiber	26.2	31.5	33.8	31.1	21.8	25.3	32.5	30.4	36.2
Calcium	10.0	11.2	11.6	9.3	11.0	11.2	8.8	9.1	11.8
Phosphorous	5.1	4.9	4.9	4.6	4.9	4.1	3.8	5.4	3.9
Gross energy (MJ/kg DM)	19.6	19.3	19.3	19.3	19.5	19.6	20.0	19.9	19.5

^1^ Composition per kg premix: 1,000,000 IU vitamin A, 400,000 IU vitamin D3, 2000 mg vitamin E, 400 mg vitamin K, 300 mg vitamin B1, 750 mg vitamin B2, 450 mg vitamin B6, 2250 µg vitamin B12, 6900 mg nicotinic acid, 1950 mg pantothenic acid, 195,000 µg folic acid and 12,000 µg biotin; 1680 mg Fe, 8000 mg Zn, 10,000 mg Mn, 1200 mg Cu, 100 mg I, 25 mg Se. ^2^ Biolys^®^ Feed Grade 54.6% Lysine (Evonik); ^3^ monensin (Elanco); ^4^ 6-phytase derived from *E. coli* (Huvepharma); ^5^ with acid hydrolysis. CON, control; SL15, substitution level 15% CP; SL30, substitution level 30% CP.

**Table 3:** Summarized performance results of broiler chickens in starter, grower, and finisher phases as well as across the complete experiment. Data are given as least-squares means with standard error.

Diets	CON	SL15	SL30	SE	*p*-Value
Starter					
Body weight d1, g	40.2	40.4	40.2	0.441	0.258
Body weight d14, g	502 ^a^	491 ^a^	468 ^b^	4.180	<0.001
Average daily feed intake, g/bird/d	31.5 ^b^	37.7 ^a^	38.0 ^a^	1.317	0.006
Average daily gain, g/d	33.0 ^a^	32.1 ^a^	30.6 ^b^	0.301	<0.001
FCR ^1^, g/g	0.955 ^b^	1.173 ^a,b^	1.241 ^a^	0.039	<0.001
Mortality, *n*	0	0	3		
Grower					
Body weight d28, g	1638 ^a^	1634 ^a^	1465 ^b^	25.58	<0.001
Average daily feed intake, g/bird/d	110 ^a^	107 ^a^	99.2 ^b^	2.364	0.029
Average daily gain, g/d	81.2 ^a^	81.7 ^a^	71.2 ^b^	1.715	<0.001
FCR, g/g	1.351	1.305	1.398	0.036	0.229
Mortality, *n*	0	1	4		
Finisher					
Body weight d35, g	2432 ^a^	2435 ^a^	2244 ^b^	45.46	<0.001
Average daily feed intake, g/bird/d	174 ^a^	172 ^a,b^	159 ^b^	4.436	0.025
Average daily gain, g/d	113	114	111	3.256	0.586
FCR, g/g	1.539 ^y^	1.510 ^y,z^	1.433 ^z^	0.040	0.079
Mortality, *n*	1	0	5		
Overall phase					
Average daily feed intake, g/d	91.3	92.2	86.7	1.795	0.093 ^2^
Average daily gain, g/d	68.3 ^a^	68.4 ^a^	63.0 ^b^	1.296	0.001
FCR, g/g	1.337	1.348	1.379	0.029	0.442
Mortality, *n*	1	1	12		

CON, control; SL15, substitution level 15% CP; SL30, substitution level 30% CP; SE, standard error. ^1^ Feed conversion ratio not corrected for mortality. ^2^ Post hoc Tukey test *p* > 0.102. ^a,b^ Indicate differences between CON, SL15, and SL30 (*p* ≤ 0.05); ^y,z^ indicate differences by trend between CON, SL15, and SL30 (0.05 < *p* ≤ 0.10).

**Table 4:** Carcass traits and weight of giblets of broilers fed increasing concentrations of HI larvae meal. Data are given as least-squares means with standard error.

Treatment Group	CON	SL15	SL30	SE	*p*-Value
Eviscerated carcass, g	2017 ^a^	2040 ^a^	1826 ^b^	43.88	<0.001
Eviscerated carcass chilled, g	1973 ^a^	1994 ^a^	1780 ^b^	43.60	<0.001
Dressing, %	76.1 ^y,z^	78.4 ^y^	74.9 ^z^	1.338	0.075
Carcass for grilling, g	1786 ^a^	1813 ^a^	1610 ^b^	39.19	<0.001
Abdominal fat, g	23.6 ^a^	21.5 ^a^	17.1 ^b^	1.110	<0.001
Heart, g	12.9	13.2	12.5	0.644	0.588
Liver, g	51.9 ^b^	55.8 ^a^	52.2 ^b^	1.771	0.030
Gizzard, g	27.0 ^a^	24.5 ^b^	19.5 ^c^	0.734	<0.001
Wings, g	215	205	199	6.011	0.150
Legs, g	77.8	77.8	77.8	3.218	0.999
Breast, g	532 ^z^	577 ^y^	557 ^y,z^	15.75	0.057

CON, control; SL15, substitution level 15% CP; SL30, substitution level 30% CP; SE, standard error. ^a,b,c^ Indicate differences between CON, SL15, and SL30 (*p* ≤ 0.05); ^y,z^ indicate differences by trend between CON, SL15, and SL30 (0.05 < *p* ≤ 0.10).

**Table 5:** Apparent ileal digestibility (%) of crude protein and amino acids of broilers fed increasing concentrations of HI larvae meal. Data are given as least-squares means with standard error.

Treatment Group	CON	SL15	SL30	SE	*p*-Value
CP	79.6 ^a^	75.7 ^a,b^	74.3 ^b^	1.154	0.014
Indispensable amino acids					
Arginine	87.6	85.3	86.0	1.004	0.242
Histidine	83.4 ^a^	80.1 ^a,b^	79.1 ^b^	1.096	0.031
Isoleucine	78.1 ^a^	75.7 ^a^	69.5 ^b^	1.747	0.008
Leucine	81.9 ^a^	77.4 ^b^	76.5 ^b^	1.229	0.016
Lysine	82.8	79.4	80.1	1.282	0.152
Methionine	88.4	86.4	86.7	1.011	0.333
Phenylalanine	83.7	80.9	80.5	1.157	0.143
Threonine	75.2	72.6	72.0	1.375	0.140
Valine	79.2	76.8	76.8	1.349	0.315
Dispensable amino acids					
Alanine	80.7	78.7	79.7	1.404	0.565
Asparagine	80.9 ^a^	77.5 ^a,b^	76.5 ^b^	1.186	0.044
Cysteine	74.6 ^a^	70.2 ^b^	67.9 ^b^	1.496	0.007
Glycine	76.4 ^a^	72.6 ^a,b^	71.5 ^b^	1.387	0.045
Glutamine	86.5 ^y^	83.7 ^y,z^	83.1 ^z^	0.976	0.068
Proline	82.6	81.3	79.9	1.092	0.214
Serine	81.3 ^a^	77.9 ^a,b^	77.0	1.192	0.041
Methionine + Cysteine	83.0	80.3	79.8	1.162	0.106

CON, control; SL15, substitution level 15% CP; SL30, substitution level 30% CP; SE, standard error. ^a,b^ Indicate differences between CON, SL15, and SL30 (*p* ≤ 0.05); ^y,z^ indicate differences by trend between CON, SL15, and SL30 (0.05 < *p* ≤ 0.10).

**Table 6:** Intestinal morphometric indices of broilers fed increasing concentrations of HI larvae meal. Data are given as least-squares means with standard error.

	Treatment Group				
	CON	SL15	SL30	SE	*p*-Value
Jejunum					
Villus height, µm	1006	988	966	69.04	0.864
Villus width, µm	175 ^z^	205 ^y^	200 ^y,z^	12.19	0.083
Villus area, mm^2^	0.18	0.20	0.19	0.017	0.541
Crypt depth, µm	141	162	166	9.413	0.128
Villus height/crypt depth	7.55	6.73	6.33	0.487	0.165
Goblet cells villus, n/200 µm	12.1	12.1	11.7	0.515	0.831
Mucosa, µm	1163	1170	1150	74.06	0.965
Submucosa, µm	41.4	44.2	43.3	2.395	0.686
Tunica muscularis circular layer, µm	161	162	175	10.66	0.538
Ileum					
Villus height, µm	621	637	603	39.08	0.808
Villus width, µm	152	148	158	9.392	0.746
Villus area, mm^2^	0.09	0.10	0.09	0.076	0.960
Crypt depth, µm	177	180	171	11.72	0.851
Villus height/crypt depth	3.92	3.91	3.80	0.295	0.937
Goblet cells villus, n/200 µm	14.1	14.5	13.9	0.731	0.822
Mucosa, µm	813	828	778	44.36	0.715
Submucosa, µm	47.6	49.4	47.7	2.836	0.880
Tunica muscularis circular layer, µm	333	335	316	25.48	0.838

CON, control; SL15, substitution level 15% CP; SL30, substitution level 30% CP; SE, standard error. ^y,z^ Indicate differences by trend between CON, SL15, and SL30 (0.05 < *p* ≤ 0.10).

**Table 7:** Microbial metabolites (on fresh matter basis) in cecal digesta of broilers fed increasing concentrations of HI larvae meal. Data are given as least-squares means with standard error.

	Treatment Group				
	CON	SL15	SL30	SE	*p*-Value
Agmatine, mg/kg	765	701	400	146.8	0.209
Ethanolamine, mg/kg	50.4	49.1	33.2	15.59	0.682
Methylamine, mg/kg	9.6	13.5	9.7	3.036	0.552
Putrescine, mg/kg	9.00	7.03	5.22	2.216	0.411
Cadaverine, mg/kg	15.8	8.01	6.25	3.508	0.142
Spermidine, mg/kg	252	245	170	46.48	0.400
Spermine, mg/kg	14.9	18.3	18.3	5.411	0.834
Ammonia (µmol/g)	17.2 ^a^	16.1 ^a^	10.7 ^b^	1.423	0.019
Lactic acid (mg/kg)	2.67	3.23	4.45	1.274	0.613

CON, control; SL15, substitution level 15% CP; SL30, substitution level 30% CP; SE, standard error. ^a,b^ Indicate differences between CON, SL15, and SL30 (*p* ≤ 0.05).

## Data Availability

Not applicable.

## References

[1] McMichael A.J., Bambrick H.J. (2005). Meat consumption trends and health: Casting a wider risk assessment net. Public Health Nutr..

[2] European Commission EU Feed Protein Balance Sheet. https://ec.europa.eu/info/sites/info/files/food-farming-fisheries/farming/documents/eu-feed-protein-balance-sheet_2017-18_en.pdf.

[3] EuropaBio The EU Protein Gap Trade Policies and GMOs. https://www.europabio.org/sites/default/files/EU_protein_GAP_WCover.pdf.

[4] van Huis A. (2013). Potential of insects as food and feed in assuring food security. Annu. Rev. Entomol..

[5] van Huis A. (2015). Edible insects contributing to food security?. Agric. Food Secur..

[6] Moula N., Detilleux J. (2019). A Meta-Analysis of the effects of insects in feed on poultry growth performances. Animals.

[7] European Commission Commission Regulation (EU) 2017/893. https://eur-lex.europa.eu/legal-content/EN/TXT/PDF/?uri=CELEX:32017R0893&from=DE.

[8] Schiavone A., de Marco M., Martínez S., Dabbou S., Renna M., Madrid J., Hernandez F., Rotolo L., Costa P., Gai F. (2017). Nutritional value of a partially defatted and a highly defatted black soldier fly larvae (*Hermetia illucens* L.) meal for broiler chickens: Apparent nutrient digestibility, apparent metabolizable energy and apparent ileal amino acid digestibility. J. Anim. Sci. Biotechnol..

[9] de Marco M., Martínez S., Hernandez F., Madrid J., Gai F., Rotolo L., Belforti M., Bergero D., Katz H., Dabbou S. (2015). Nutritional value of two insect larval meals (*Tenebrio molitor* and *Hermetia illucens*) for broiler chickens: Apparent nutrient digestibility, apparent ileal amino acid digestibility and apparent metabolizable energy. Anim. Feed Sci. Technol..

[10] Abd El-Hack M.E., Shafi M.E., Alghamdi W.Y., Abdelnour S.A., Shehata A.M., Noreldin A.E., Ashour E.A., Swelum A.A., Al-Sagan A.A., Alkhateeb M. (2020). Black soldier fly (*Hermetia illucens*) meal as a promising feed ingredient for poultry: A comprehensive review. Agriculture.

[11] Matin N., Utterback P., Parsons C.M. (2021). True metabolizable energy and amino acid digestibility in black soldier fly larvae meals, cricket meal, and mealworms using a precision-fed rooster assay. Poult. Sci..

[12] Danieli P.P., Lussiana C., Gasco L., Amici A., Ronchi B. (2019). The effects of diet formulation on the yield, proximate composition, and fatty acid profile of the black soldier fly (*Hermetia illucens* L.) prepupae intended for animal feed. Animals.

[13] Barroso F.G., Sánchez-Muros M.-J., Segura M., Morote E., Torres A., Ramos R., Guil J.-L. (2017). Insects as food: Enrichment of larvae of *Hermetia illucens* with omega 3 fatty acids by means of dietary modifications. J. Food Compos..

[14] Liu X., Chen X., Wang H., Yang Q., Ur Rehman K., Li W., Cai M., Li Q., Mazza L., Zhang J. (2017). Dynamic changes of nutrient composition throughout the entire life cycle of black soldier fly. PLoS ONE.

[15] Barragan-Fonseca K.B., Dicke M., van Loon J. (2017). Nutritional value of the black soldier fly (*Hermetia illucens* L.) and its suitability as animal feed—A review. J. Insects Food Feed.

[16] Cullere M., Tasoniero G., Giaccone V., Miotti-Scapin R., Claeys E., de Smet S., Dalle Zotte A. (2016). Black soldier fly as dietary protein source for broiler quails: Apparent digestibility, excreta microbial load, feed choice, performance, carcass and meat traits. Animal.

[17] Dabbou S., Gai F., Biasato I., Capucchio M.T., Biasibetti E., Dezzutto D., Meneguz M., Plachà I., Gasco L., Schiavone A. (2018). Black soldier fly defatted meal as a dietary protein source for broiler chickens: Effects on growth performance, blood traits, gut morphology and histological features. J. Anim. Sci. Biotechnol..

[18] Schiavone A., Dabbou S., Petracci M., Zampiga M., Sirri F., Biasato I., Gai F., Gasco L. (2019). Black soldier fly defatted meal as a dietary protein source for broiler chickens: Effects on carcass traits, breast meat quality and safety. Animal.

[19] Velten S., Neumann C., Bleyer M., Gruber-Dujardin E., Hanuszewska M., Przybylska-Gornowicz B., Liebert F. (2018). Effects of 50 percent substitution of soybean meal by alternative proteins from *Hermetia illucens* or *Spirulina platensis* in meat-type chicken diets with graded amino acid supply. Open J. Anim. Sci..

[20] Janssen R.H., Vincken J.-P., van den Broek L.A.M., Fogliano V., Lakemond C.M.M. (2017). Nitrogen-to-Protein conversion factors for three edible insects: *Tenebrio molitor*, *Alphitobius diaperinus*, and *Hermetia illucens*. J. Agric. Food Chem..

[21] Yamauchi K. (2007). Review of a histological intestinal approach to assessing the intestinal function in chickens and pigs. Anim. Sci. J..

[22] Wang J.X., Peng K.M. (2008). Developmental morphology of the small intestine of African ostrich chicks. Poult. Sci..

[23] Günther C., Neumann H., Neurath M.F., Becker C. (2013). Apoptosis, necrosis and necroptosis: Cell death regulation in the intestinal epithelium. Gut.

[24] Apajalahti J., Vienola K. (2016). Interaction between chicken intestinal microbiota and protein digestion. Anim. Feed Sci. Technol..

[25] Qaisrani S.N., van Krimpen M.M., Kwakkel R.P., Verstegen M., Hendriks W.H. (2015). Dietary factors affecting hindgut protein fermentation in broilers: A review. World Poultry Sci. J..

[26] Aviagen Ross 308 Broiler Nutrition Specifications. http://en.aviagen.com/assets/Tech_Center/Ross_Broiler/RossBroilerNutritionSpecs2019-EN.pdf.

[27] Naumann C., Bassler R. (2012). Die chemische Untersuchung von Futtermitteln, 3. Aufl..

[28] Hansen B. (1989). Determination of nitrogen as elementary N, an alternative to Kjeldahl. Acta. Agric. Scand..

[29] Licitra G., Hernandez T.M., van Soest P.J. (1996). Standardization of procedures for nitrogen fractionation of ruminant feeds. Anim. Feed Sci. Technol..

[30] Leone J.L. (1973). Collaborative study of the quantitative determination of titanium dioxide in cheese. J. Assoc. Off. Anal. Chem..

[31] Figueiredo-Silva C., Lemme A., Sangsue D., Kiriratnikom S. (2015). Effect of DL-methionine supplementation on the success of almost total replacement of fish meal with soybean meal in diets for hybrid tilapia (*Oreochromis niloticus* × *Oreochromis mossambicus*). Aquac. Nutr..

[32] Saarinen M.T. (2002). Determination of biogenic amines as dansyl derivatives in intestinal digesta and feces by reversed phase HPLC. Chromatographia.

[33] Pryce J.D. (1969). A modification of the Barker-Summerson method for the determination of lactic acid. Analyst.

[34] Marono S., Loponte R., Lombardi P., Vassalotti G., Pero M.E., Russo F., Gasco L., Parisi G., Piccolo G., Nizza S. (2017). Productive performance and blood profiles of laying hens fed *Hermetia illucens* larvae meal as total replacement of soybean meal from 24 to 45 weeks of age. Poult. Sci..

[35] Rothstein Née Velten S. (2019). Ernährungsphysiologische Bewertung von Teilentfettetem Larvenmehl der Schwarzen Soldatenfliege (*Hermetia illucens*) für Den Einsatz in Ressourcenschonenden Ernährungskonzepten der Schweine- und Hähnchenmast. Doctoral Dissertation.

[36] Gillette K., Thomas D.K., Bellingham W.P. (1983). A parametric study of flavoured food avoidance in chicks. Chem. Senses.

[37] Pousga S., Boly H., Ogle B. (2005). Choice feeding of poultry: A review. Livest. Res. Rural Dev..

[38] Pieterse E., Erasmus S.W., Uushona T., Hoffman L.C. (2019). Black soldier fly (*Hermetia illucens*) pre-pupae meal as a dietary protein source for broiler production ensures a tasty chicken with standard meat quality for every pot. J. Sci. Food Agric..

[39] Altmann B., Neumann C., Velten S., Liebert F., Mörlein D. (2018). Meat quality derived from high inclusion of a micro-alga or insect meal as an alternative protein source in poultry diets: A pilot study. Foods.

[40] Rinaudo M. (2006). Chitin and chitosan: Properties and applications. Prog. Polym. Sci..

[41] Hossain S.M., Blair R. (2007). Chitin utilisation by broilers and its effect on body composition and blood metabolites. Br. Poult. Sci..

[42] Tabata E., Kashimura A., Kikuchi A., Masuda H., Miyahara R., Hiruma Y., Wakita S., Ohno M., Sakaguchi M., Sugahara Y. (2018). Chitin digestibility is dependent on feeding behaviors, which determine acidic chitinase mRNA levels in mammalian and poultry stomachs. Sci. Rep..

[43] Finke M.D. (2007). Estimate of chitin in raw whole insects. Zoo Biol..

[44] van Soest P.J. (1994). Nutritional Ecology of the Ruminant.

[45] Nery J., Gasco L., Dabbou S., Schiavone A. (2018). Protein composition and digestibility of black soldier fly larvae in broiler chickens revisited according to the recent nitrogen-protein conversion ratio. J. Insects Food Feed.

[46] Ravindran V. (2016). Feed-induced specific ileal endogenous amino acid losses: Measurement and significance in the protein nutrition of monogastric animals. Anim. Feed Sci. Technol..

[47] Yamauchi K. (2002). Review on chicken intestinal villus histological alterations related with intestinal function. J. Poult. Sci..

[48] Chu X., Li M., Wang G., Wang K., Shang R., Wang Z., Li L. (2020). Evaluation of the low inclusion of full-fatted *Hermetia illucens* larvae meal for layer chickens: Growth performance, nutrient digestibility, and gut health. Front. Vet. Sci..

[49] Biasato I., Ferrocino I., Biasibetti E., Grego E., Dabbou S., Sereno A., Gai F., Gasco L., Schiavone A., Cocolin L. (2018). Modulation of intestinal microbiota, morphology and mucin composition by dietary insect meal inclusion in free-range chickens. BMC Vet. Res..

[50] Cutrignelli M.I., Messina M., Tulli F., Randazzo B., Olivotto I., Gasco L., Loponte R., Bovera F. (2018). Evaluation of an insect meal of the Black Soldier Fly (*Hermetia illucens*) as soybean substitute: Intestinal morphometry, enzymatic and microbial activity in laying hens. Res. Vet. Sci..

[51] Qaisrani S.N., Moquet P.C.A., van Krimpen M.M., Kwakkel R.P., Verstegen M.W.A., Hendriks W.H. (2014). Protein source and dietary structure influence growth performance, gut morphology, and hindgut fermentation characteristics in broilers. Poult. Sci..

[52] Bryan D.D.S.L., Abbott D.A., van Kessel A.G., Classen H.L. (2020). The influence of indigestible protein on broiler digestive tract morphology and caecal protein fermentation metabolites. J. Anim. Physiol. Anim. Nutr..

[53] Pieper R., Kröger S., Richter J.F., Wang J., Martin L., Bindelle J., Htoo J.K., von Smolinski D., Vahjen W., Zentek J. (2012). Fermentable fiber ameliorates fermentable protein-induced changes in microbial ecology, but not the mucosal response, in the colon of piglets. J. Nutr..

